# Robust serotonin activation of the kisspeptin GnRH pulse generator in male and female mice

**DOI:** 10.1210/endocr/bqag034

**Published:** 2026-03-30

**Authors:** Paul G Morris, Xinhuai Liu, Emily Birt, Szilvia Vas, Miguel Ruiz Cruz, Danielle Schafer, H James McQuillan, Allan E Herbison

**Affiliations:** Department of Physiology, Development and Neuroscience, Cambridge CB2 3EG, UK; Centre for Neuroendocrinology and Department of Physiology, University of Otago School of Biomedical Sciences, Dunedin 9054, New Zealand; Department of Physiology, Development and Neuroscience, Cambridge CB2 3EG, UK; Department of Physiology, Development and Neuroscience, Cambridge CB2 3EG, UK; Department of Physiology, Development and Neuroscience, Cambridge CB2 3EG, UK; Centre for Neuroendocrinology and Department of Physiology, University of Otago School of Biomedical Sciences, Dunedin 9054, New Zealand; Centre for Neuroendocrinology and Department of Physiology, University of Otago School of Biomedical Sciences, Dunedin 9054, New Zealand; Department of Physiology, Development and Neuroscience, Cambridge CB2 3EG, UK; Centre for Neuroendocrinology and Department of Physiology, University of Otago School of Biomedical Sciences, Dunedin 9054, New Zealand

**Keywords:** GnRH, kisspeptin, GCaMP, 5-HT, luteinizing hormone, pulsatility

## Abstract

Serotonin neurons are thought to exert a modulatory influence on the secretion of the gonadotropin hormones in mammals, but their mechanism of action remains unclear. We examined here the potential role of serotonin neurons in modulating the activity of the gonadotropin-releasing hormone (GnRH) pulse generator formed by the arcuate nucleus kisspeptin (ARN^KISS^) neurons. Acute brain slice electrophysiology revealed that ∼60% of ARN^KISS^ neurons in diestrous female mice were activated by serotonin while less than 10% were inhibited. Pharmacological studies indicated that combinatorial patterns of 5-HT receptor subtype activation were likely responsible for the excitatory actions. The role of serotonin in ARN^KISS^ neuron synchronization behavior was assessed using GCaMP imaging in acute brain slices from diestrous female and male mice. In both sexes, serotonin-evoked potent recurring bouts of synchronization activity amongst ARN^KISS^ neurons. To evaluate the impact of serotonin in vivo, we used “fluidic” GCaMP fiber photometry in which serotonin was infused directly into the ARN while recording the ARN^KISS^ neuron population activity in freely behaving diestrous female mice. In all cases, the infusion of serotonin evoked a robust ARN^KISS^ neuron synchronization episode. These data demonstrate that serotonin exerts a direct, predominantly stimulatory action on ARN^KISS^ neuron pulse generator through a variety of 5-HT receptors. Serotonergic inputs appear to provide a potent synchronizing influence on the ARN^KISS^ neuron population and suggest considerable potential for 5-HT to control the frequency of pulsatile reproductive hormone secretion in mice and likely other mammals.

Serotonergic neurons project widely in the forebrain where they modulate the activity of most neural networks (1). Although there are many accounts of abnormal sexual functioning and subfertility in individuals taking selective serotonin reuptake inhibitors (SSRI) (2), it remains unclear how serotonin modulates the neural networks controlling fertility. With respect to pulsatile reproductive hormone secretion, the activation of serotonin receptors throughout the brain by intracerebroventricular (ICV) administration has been found to inhibit pulsatile luteinizing hormone (LH) secretion in ovariectomized (OVX) animals but increase secretion in the intact or estrogen-replaced OVX state (3, 4). This biphasic pattern of behavior is observed for many transmitters (5) and may result from state-dependent activation of the GnRH pulse generator (6). As such, most evidence is compatible with serotonin exerting a facilitatory influence on pulsatile hormone release. Serotonin increases gonadotropin-releasing hormone (GnRH) pulse frequency when measured from hypothalamic chunks obtained from estrogen-replaced OVX rats (7) and the broad-spectrum serotonin receptor antagonist methysergide abolishes pulsatile LH release in the OVX guinea-pig (8). Furthermore, administration of the SSRI fluoxetine can re-activate pulsatile LH release or estrous cycles in stressed rats and mice (9, 10).

One pathway through which serotonin neurons may influence reproductive hormone secretion involves the direct modulation of the GnRH pulse generator. This is formed by the arcuate nucleus kisspeptin (ARN^KISS^) neuron population that project to GnRH neurons and drive the episodic release of GnRH into the pituitary portal circulation to generate pulsatile LH secretion (11, 12). To date, both gene profiling and electrophysiological studies indicate that serotonin (5-HT) can directly modulate the activity of ARN^KISS^ neurons. Brain slice electrophysiological experiments in mice have shown that serotonin can activate ARN^KISS^ neuron firing and that this effect is at least partly mediated by 5-HT_4_ receptors (13). In contrast, only the Htr2c transcript was detected in ARN^KISS^ neurons in OVX rats (9). Further, the ICV administration of serotonin in the goat was found to drive a volley of multi-unit activity in the ARN, believed to represent ARN^KISS^ neurons, and this was blocked by a 5-HT_2c_ antagonist (9).

While there is growing evidence that serotonergic neurons can modulate the activity of the kisspeptin pulse generator, discrepancies exist in the reported literature and the precise sites at which serotonin acts to modulate the activity of these cells is unclear. One key question is whether serotonergic transmission at the level of the ARN^KISS^ neuron impacts on their synchronization behavior. We aimed here to provide an account of serotonin actions on individual ARN^KISS^ neurons using brain slice electrophysiology and on synchronized population activity of ARN^KISS^ neurons using both ex vivo and in vivo GCaMP calcium imaging.

## Materials and methods

### Mice

For brain slice electrophysiology studies, Kiss1-GFP mice were generated and used at the University of Otago as reported previously (14) by crossing Kiss1-Cre^+/−^ mice (15) with a homozygous Rosa26-CAGS-τGFP reporter line (16) to generate mixed background 129S6Sv/Ev C57BL6 Kiss1-Cre,Rosa26 τGFP-lox-STOP-lox mice.

For brain slice GCaMP imaging and whole-cell electrophysiology experiments, Kiss1-GCaMP6 mice were generated and used at the University of Cambridge as detailed previously (17) by crossing the 129S6Sv/Ev C57BL/6 Kiss1^Cre/+^ (18) and C57BL/6 Ai162 (TIT2L-GC6s-ICL-tTA2)-D Cre-dependent GCaMP6s line (JAX stock #031562) (19).

For in vivo GCaMP fiber photometry, Kiss1-GCaMP6 mice were generated and used at the University of Otago as detailed previously (20) by giving intracerebral AAV injections to 129S6Sv/Ev C57BL/6 Kiss1^Cre/+^ (18) mice.

Unless specified below, all mice were group-housed in cages with environmental enrichment under conditions of controlled temperature (22 ± 2 °C) and lighting (12-hour light/12-hour dark cycle; lights on at 6:00 or 7:00 hours) with ad libitum access to food (Otago: Teklad Global 18% Protein Rodent Diet 2918, Envigo, Huntingdon, UK; Cambridge: RM1-P, SDS, UK) and water. Vaginal cytology was used to follow mouse estrous cycles and select mice for experimentation on diestrus. All experimental protocols were approved by the University of Otago Animal Ethics Committee or the University of Cambridge Animal Welfare and Ethical Review Body, UK (P174441DE). Mice were 10-22 weeks old at the time of experimentation.

### Brain slice electrophysiology

ARN^KISS^ neuron brain slice electrophysiology was undertaken as reported previously (21). In brief, Kiss1-GFP mice were euthanized by cervical dislocation, decapitated, and the brain quickly removed to prepare 250 μm-thick coronal brain slices containing the ARN on a vibratome (Leica VT1000S) in ice-cooled (∼2 °C) “slicing” artificial cerebrospinal fluid (aCSF; [in mM] 75 NaCl, 2.5 KCl, 25 D-glucose, 75 sucrose, 15 NaHCO_3_, 20 HEPES, 0.25 CaCl_2_, and 6 MgCl_2_, equilibrated with carbogen [95% O_2%_ and 5% CO_2_]). Brain slices were then transferred to aCSF containing (in mM) 118 NaCl, 3 KCl, 11 D-glucose, 25 NaHCO_3_, 10 HEPES, 2.5 CaCl_2_, and 1.2 MgCl_2_ and equilibrated with carbogen at 30 ± 1 °C for >1 hour before being placed in a recording chamber perfused at 2-3 mL/min. Loose-seal cell-attached recordings (10-30 MΩ) were made from GFP-expressing ARN^KISS^ neurons visualized through an upright microscope fitted for epifluorescence (Olympus, Tokyo, Japan). Cells were identified by brief fluorescence illumination and approached using Nomarski differential interference contrast optics. Recording electrodes (3.5-5.0 MΩ) were pulled from borosilicate capillaries (Warner Instruments, Hamden, CT, USA) with a horizontal puller (Sutter Instruments, Novato, CA, USA) and filled with (in mM) 145 NaCl, 3 KCl, 10 HEPES, 2.5 CaCl_2_, and 1.2 MgCl_2_, (pH 7.35 adjusted by KOH, ∼290 mOsmol).

Loose seals were formed, and recordings of action current signals were undertaken in the voltage-clamp mode with a 0-mV voltage command. Signals were amplified with a Multiclamp 700B amplifier (Molecular Devices, USA) and sampled on-line using a Digidata 1440A interface (Molecular Devices, USA). Signals were filtered with the Bessel filter of the Multiclamp 700B (3 kHz) before being digitized at a rate of 10 kHz. Acquisition and subsequent analysis of the acquired data were performed with the Clampex 10 suite of software (Molecular Devices, USA) and Origin Pro 7.5 (OriginLab Corporation, USA). Once a stable cell-attached voltage-clamp recording with 0 pA holding current was achieved, the aCSF was switched to 1 containing amino acid receptor antagonists (5 µM GABAzine, 50 µM D-(-)-2-Amino-5-phosphonopentanoic acid (DAP5), and 10 µM 6-cyano-7-nitroquinoxaline-2,3-dione (CNQX)). Serotonin (10-60 µM) was then puffed immediately above the slice surface and about 40 µM away from the recorded cell using a microelectrode driven by a pneumatic picopump (WPI, USA) with a low pressure (∼2PSI). All other drugs were bath-applied by adding into recording aCSF at the concentrations indicated. Neurons were considered to have been excited or inhibited if their firing rate changed by >50%.

For whole-cell recordings, pipettes (∼2.5-3.5 MΩ) were filled with an intracellular solution composed of (mM): potassium gluconate 135; KCl 10; EGTA 5; HEPES 10; CaCl2 0.1; Mg-ATP 4; Na-GTP 0.4; with pH at 7.3 and filtered at 0.2 µm. The resting membrane voltage was recorded, and 5-HT (30 µM) applied for 90 seconds via bath perfusion. Mean voltage was recorded between 2 and 1 minute before 5-HT application, the 2 minutes period following 5-HT application, then 18-20 minutes following application (wash). Tetrodotoxin (TTX, 1 µM), CNQX, 20 µM, DAP5 (20 µM), and bicuculline (BIC, 40 µM) were all applied continuously throughout these recordings.

Stock solutions of GABAzine (SR95531, 5 mM, Tocris, UK), CNQX (10 mM, Tocris, UK), and DAP5, (50 mM, Tocris, UK) were prepared with Milli-Q water or NaOH for DAP5. The stock solutions of serotonin (30 or 60 mM, Sigma, NZ & UK) and zacopride (10 mM, Tocris, UK) were diluted in Milli-Q water. TTX stock (2 mM, Tocris, UK) was prepared in Milli-Q water pH adjusted to 4.8 using HCl. Stock solutions of SB228357 (100 mM), CNQX (40 mM, Tocris, UK), and BIC (100 mM, Tocris, UK) were prepared in DMSO. All stock solutions were kept at −20 °C.

### Ex vivo brain slice GCaMP imaging

Brain slices were prepared exactly as reported previously (17). Kiss1-GCaMP6 mice were anesthetized using isoflurane, decapitated, and the brain removed into oxygenated, ice-cold slicing solution composed of (mM): NaCl 52.5; sucrose 100; glucose 25; NaHCO_3_ 25; KCl 2.5; CaCl_2_ 1; MgCl_2_ 5; NaH_2_PO_4_ 1.25; kynurenic acid 0.1 (95% O_2_/5% CO_2_). Coronal slices containing the ARN were prepared at 280 µm thickness using a VT1200S tissue slicer (Leica Biosystems UK) before being transferred to a submersion chamber containing an oxygenated (95% O_2_/5% CO_2_) aCSF recording solution composed of (mM): NaCl 124; glucose 30; NaHCO_3_ 25; KCl 3.5; CaCl_2_ 1.5; MgCl_2_ 1; NaH_2_PO_4_ 0.5 and incubated at 30 °C for 1-5 hours prior to use.

Slices containing the ARN were transferred to the stage of an Olympus BX51WI upright microscope with differential interference contrast optics, and constantly perfused with oxygenated aCSF at 30 ± 1 °C. Fluctuations in the intracellular calcium concentration of ARN^KISS^ neurons were estimated by recording their GCaMP6s fluorescence as previously described (17). ImageJ (v1.53c) was used to obtain mean fluorescence intensities over the image time series: active cell somata were selected manually as regions of interest (ROIs), and for each, the mean fluorescence values of a nearby background ROI was subtracted. Fluorescence intensity data and all metrics described below were analyzed using custom Python scripts. The change in fluorescence (ΔF/F) was calculated and individual calcium events in cells and population “miniature” synchronization events (mSEs) were registered as previously described (17). Briefly, an mSE is recorded if the peak of calcium events from at least 2 neurons occurs within 10 seconds of each other. Event and mSE rates are presented as “per cell, per hour”, thereby controlling for the variation in the number of neurons being recorded in each brain slice, and for mSEs this reflects the rate at which a single neuron contributes to synchronized activity.

Within each experiment, a predrug baseline was obtained, followed by a 3-minute application of 5-HT, and an extended wash period. 5-HT elicited ongoing network activity, therefore the “post-5-HT” analysis period was set as 15-25 minutes to capture this. In the spontaneously active slice preparation, individual neurons were defined as “5-HT-responsive” if the drug epoch produced at least a 3-fold increase in event rate to ≥6 events/hour or increased from zero to ≥6 events/hour. This increase was calibrated based on the data and captures large increases in activity whilst filtering out spurious larger fold-changes caused by near-zero starting values.

### In vivo microfluidic GCaMP photometry experiments

Mice were prepared for ARN^KISS^ neuron GCaMP fiber photometry as previously reported (20, 22). In brief, adult female Kiss1-Cre mice were given meloxicam (5 mg/kg, sc.), buprenorphine (0.05 mg/kg, sc.) and dexamethasone (10 mg/kg, sc.), and anesthetized with isoflurane (1%-2%, 1 L/min) and placed in a stereotaxic frame. Mice received a unilateral 1 μL injection of AAV9-CAG-FLEX-GCaMP6s-WPRE-SV40 (1.3 × 10^13^ GC/mL, University of Pennsylvania Vector Core) into the ARN followed by implantation of an optical fiber (400 µm diameter; 0.48 NA) and an attached fluid injection port (25G, 485 μm outside diameter; Doric Lenses, Quebec, Canada) immediately above the middle/caudal ARN. Meloxicam (5 mg/kg) was administered orally for postoperative pain relief. Mice received daily handling and habituation to the photometry recording procedure over 4-6 weeks before experimentation and were single housed until the end of the study.

Regularly cycling mice, as determined by vaginal lavage and cytology (23), were investigated between 10 Am and 4 Pm on the diestrous stage of the cycle. Following a period of baseline photometry recording, a stainless-steel fluid injector (Doric Lenses, Quebec, Canada) was inserted into the infusion cannula of the headgear as described earlier (17, 24). Injector dummies were removed from the fluidic port immediately prior to insertion of the injector and replaced immediately following completion of the experiment. Infusions of 1 μL saline or 10 μM 5-HT dissolved in 1 μL saline at a rate of either 0.1 or 0.5 μL/min were delivered > 20 minutes after inserting the injector using preloaded polyethylene tubing connected to a 1 μL Hamilton syringe and a Nanojet stereotaxic syringe pump (Chemyx USA). Each mouse received at least 1 infusion of saline or 5-HT at least 2 weeks apart. Some mice were given a second injection of 5-HT at least 30 minutes after the first.

### Bioinformatic analysis of RNAseq data to assess serotonin receptor mRNA expression in ARN kisspeptin neurons

FASTQ files containing raw sequences from ARN^KISS^ neurons from 2 publicly available datasets (13, 25) and unpublished data from the Herbison laboratory were first quality- and adapter-trimmed using Trim Galore v0.6.7. Ribosomal RNA reads were removed using SortMeRNA v4.3.4 using the eukaryotic Rfam v14.9 database. Reads were then aligned to the mouse GRCm39 genome with STAR v2.7.10a. Gene-level counts were obtained with the FeatureCounts function from the Rsubread v2.0.0 and were subsequently normalized to transcripts per million (TPM). Expression values for serotonin receptors Htr1a, Htr1b, Htr1d, Htr2a, Htr2b, Htr2c, Htr3b, Htr5a, Htr6, and Htr7 were extracted and the percentage of samples in each data set expressing each transcript calculated. To avoid direct quantitative comparisons between datasets generated using different ARN^KISS^ neuron RNA isolation strategies and sequencing conditions, receptors were then ranked from highest (1st) to lowest (10th) expression based on their TPM values within each dataset. Ranked expression values and detection percentages were visualized using a bubble plot generated in R using ggplot2, where circle size represents ranked relative expression and color indicates the percentage of detection.

## Results

### Transcriptomic analysis of 5-HT receptor transcripts in ARN^KISS^ neurons

**Figure 1:**
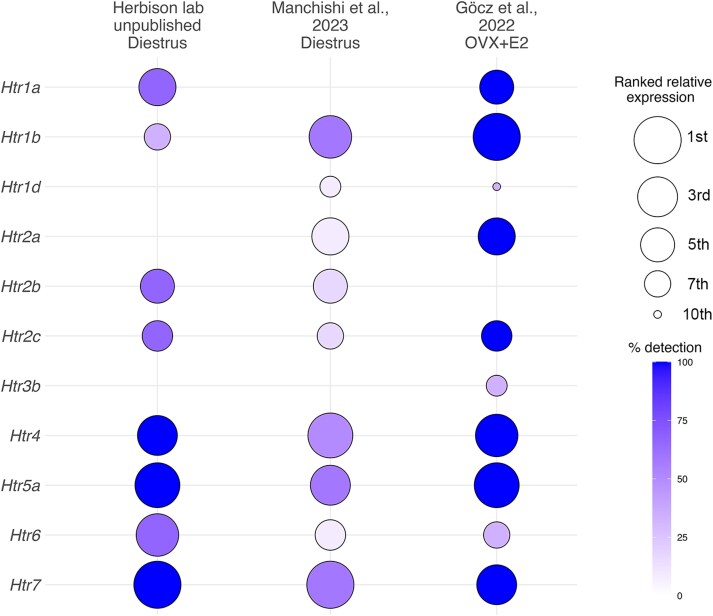
ARN^KISS^ neurons express multiple serotonin receptor subtypes across different transcriptomic datasets. Bubble plot showing the ranked expression and percentage of detection of serotonin receptor transcripts in ARN^KISS^ neurons across 3 different transcriptomic datasets. Circle size represents ranked relative expression based on TPM values within each dataset (ranked from highest to lowest), and color indicates the percentage of samples in which each transcript was detected. Data were obtained from: Herbison laboratory unpublished data (diestrus), Manchishi et al (25) (diestrus), and Gocz et al (13) (OVX + E2).

To help assess the potential 5-HT receptor subtypes expressed by ARN^KISS^ neurons we undertook a bioinformatic analysis of ARN^KISS^ neuron RNAseq data available to us. This included 2 data sets obtained from diestrous mice generated by Manchishi et al (25) and our own unpublished data, and results from OVX estradiol-treated (OVX + E2) mice generated by Gocz et al (13). This highlighted that ARN^KISS^ neurons in diestrous/OVX + E2 mice exhibit transcripts for most 5-HT receptor subtypes (Fig.1). The Htr4, Htr5a, Htr6 and Htr7 transcripts were consistently detected in the majority of the sample populations and at relatively high abundance within the 5-HT transcript family (Fig.1). The 5-HT2 receptor family of transcripts (a,b,c) were also detected at modest relative expression and incidence in most data sets (Fig.1). Most variability was detected for the Htr1a,b,d mRNAs between data sets (Fig.1). Together this analysis suggests that a wide a variety of 5-HT receptors are expressed by many ARN^KISS^ neurons in diestrous with Htr4, Htr5a, Htr6 and Htr7 being the most abundant while expression of the Htr2 and Htr1 families is variable, and Htr3 is not present.

### Brain slice electrophysiology

**Figure 2:**
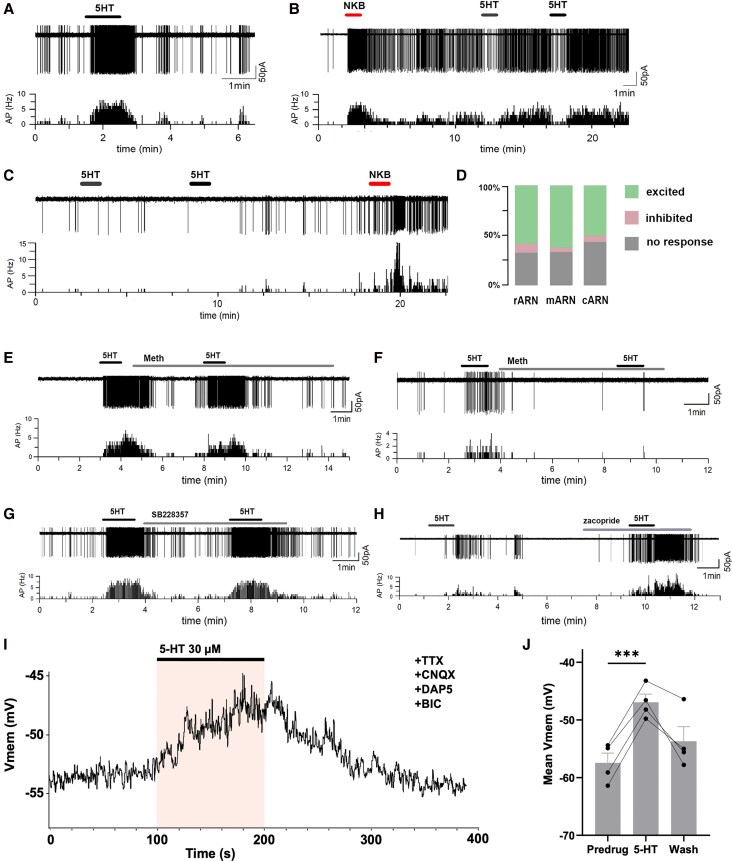
Effects of serotonin and 5-HT receptor compounds on ARN^KISS^ neuron firing in acute brain slices from diestrous female mice. (A) Robust excitatory effect of 90 seconds puff of 40 µM serotonin on firing rate of a middle ARN kisspeptin neuron. (B) Inhibitory effect of 90 seconds puff of 60 µM serotonin on firing rate of a caudal ARN kisspeptin neuron initially stimulated to fire by a 200 nM puff of NKB. (C) Rostral ARN kisspeptin neuron not responding to 60 µM puffs of serotonin but later activated by NKB. (D) Summary of percentage of rostral (rARN), middle (mARN), and caudal (cARN) kisspeptin neurons excited or inhibited by serotonin. (E) Caudal ARN kisspeptin neuron activated by serotonin (40 µM) in the absence and presence of methiothepin (100 µM). (F) Middle ARN kisspeptin neuron in which the excitatory effect of serotonin (40 µM) is blocked by methiothepin (100 µM). (G) Middle ARN kisspeptin neuron activated by serotonin (40 µM) in the absence and presence of SB228357 (100 µM). (H) Caudal ARN kisspeptin neuron in which zacopride (2 µM) facilitates serotonin excitation. (I) Whole-cell recording from an ARN^KISS^ neuron in the continuous presence of TTX, CNQX, DAP5, and bicuculline, showing depolarization during bath application of 5-HT (30 µM). (J) Summary of mean membrane potential measured predrug, during 5-HT application, and following wash (*P* < .001; *n* = 4 animals).

To assess the impact of serotonin on the ARN^KISS^ neuron population, we prepared acute brain slices from diestrous female Kiss1-GFP mice (14) and made cell-attached recordings from individual ARN^KISS^ neurons while applying small puffs of serotonin (10-60 µM) in the presence of an amino acid transmitter antagonist cocktail (GABAzine, CNQX, DAP5)(Fig. 2A-2H). Where neurons showed very little spontaneous activity, they were given a puff of 100-200 nM neurokinin B (NKB). At all rostro-caudal levels of the ARN (*N* = 35 mice), we found a predominant excitatory effect of serotonin with 58.3% (*n* = 12), 62.3% (*n* = 69) and 50.0% (*n* = 60) of rostral, middle, and caudal-located ARN^KISS^ neurons being activated (Fig. 2A and 2D) compared with 8.3%, 4.3%, and 6.3% that were inhibited, respectively (Fig. 2B and 2D). The remaining ∼30% of ARN^KISS^ neurons were unaffected by serotonin puffs (Fig. 2C and 2D).

To assess the serotonin receptors that may be underlying the excitatory effects, we preidentified ARN^KISS^ neurons excited by serotonin and then tested them again in the presence of different serotonin receptor antagonists applied through the bathing medium. Due to the large number of possible receptors (Fig. 1), we began using methiothepin (100 µM), a broad-spectrum antagonist at 5-HT_1_, 5-HT_2_, 5-HT_5_, 5-HT_6_, and 5-HT_7_ receptors. In total 23 serotonin-responsive ARN^KISS^ neurons (*N* = 23 mice) were tested and methiothepin was found to suppress 5-HT-excitation in 8 cells (35%, Fig. 2F) with no effect on serotonin excitation in the remaining 15 cells (65%; Fig. 2E). As 5-HT_5_ is exclusively inhibitory, by deduction, this suggested that 5-HT_4_ receptors may be primarily responsible for ARN^KISS^ neurons activated by serotonin while a minority were activated through 5-HT_1_, 5-HT_2_, 5-HT_6_, and/or 5-HT_7_ receptors.

Given evidence in the rat and goat that 5-HT_2_ receptors in the brain are the key subtype underlying serotonin activation of the GnRH pulse generator (9), we specifically examined the role of 5-HT_2_ receptors using the 5-HT_2A-C_ receptor antagonist SB228357. We found that SB228357 (100 µM) had no effect on the ability of puffs of serotonin to activate ARN^KISS^ neuron firing in any of the 8 cells tested (*N* = 3 mice)(Fig. 2G). This indicates that 5-HT_2_ receptors are insufficient on their own to mediate the excitatory effects of serotonin on ARN^KISS^ neurons in intact mice.

To examine the role of the 5-HT_4_ receptor, we used zacopride that is an agonist at 5-HT_4_ receptors and antagonist at 5-HT_3_ receptors (that are not expressed by ARN^KISS^ neurons). In this case, we found that zacopride (2 µM) had no immediate effect on cell firing but did greatly facilitate excitation evoked by subsequent serotonin puffs in 55% (5 of 9) of 5-HT-sensitive ARN^KISS^ neurons (*N* = 4 mice)(Fig. 2H).

To examine whether the effects of 5-HT were direct on ARN^KISS^ neurons, whole-cell recordings were performed in the presence of TTX (1 µM) and a cocktail of amino acid receptor antagonists including CNQX (20 µM), DAP5 (20 µM), and BIC (40 µM). Application of 30 µM 5-HT for 90 seconds generated a substantial depolarization (Fig. 2J; *P* < .001, *n* = 4 cells from 4 mice, *t* = 14.1, df = 3, 2-tailed paired t), with mean membrane voltage changing from −57.5 ± 1.7 mV (1 minute pre-5-HT) to −47 ± 1.1 mV (2 minutes period from 5-HT application), representing a mean voltage deflection of 10.5 ± 0.7 mV. The mean “wash” voltage recorded 18-20 minutes following the start of the 90 seconds 5-HT application was(-53.7 ± 2.5 mV (Fig. 2J).

### Brain slice calcium imaging

**Figure 3:**
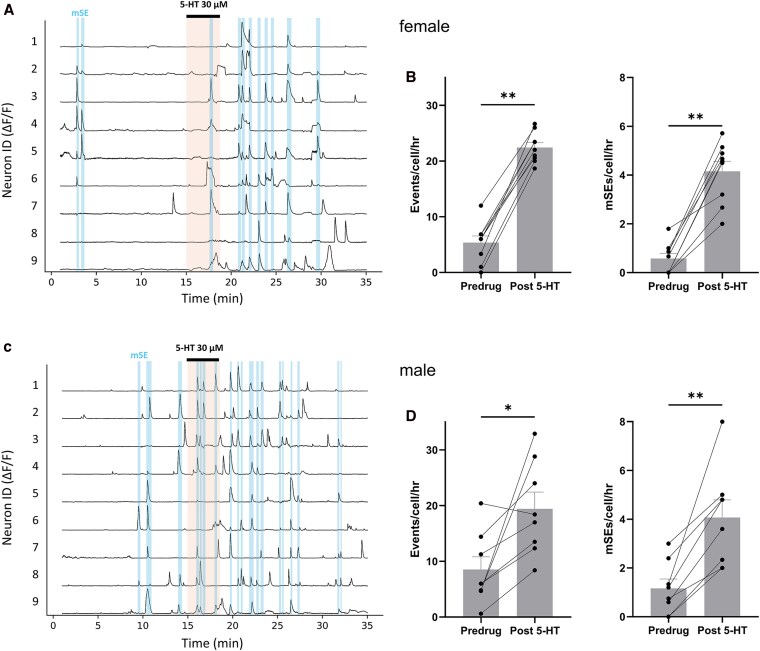
Serotonin dramatically increases rates of ARN^KISS^ neuron activation and network synchronization ex vivo. (A) Representative diestrous female GCaMP6s ΔF/F fluorescence trace recorded simultaneously from 9 ARN^KISS^ neurons in a brain slice preparation that maintains spontaneous synchronized activity within the ARN^KISS^ neuron network. Following a baseline period, the serotonin (5-HT; 30 µM) was applied for 3 minutes (orange shading) followed by a wash period. Blue shading indicates the occurrence of mini synchronization events (mSEs). (B) Histograms showing the effect of 5-HT (30 µM) on mean + SEM number of calcium events/cell/hour (**P = .0039, Wilcoxon) and mean + SEM number of mSEs/cell/hour (**P = .0039, Wilcoxon; *n* = 9 slices from *N* = 9 animals). Each datapoint represents mean population activity within a single slice/mouse, with the “post-5-HT” analysis window extending 15 minutes from the start of 5-HT application. (C). Representative male GCaMP6s ΔF/F fluorescence trace recorded simultaneously from 9 ARN^KISS^ neurons as in (A). (D) Histograms showing the effect of 5-HT (30 µM) on mean + SEM number of calcium events/cell/hour (*P = .016, Wilcoxon) and mean + SEM number of mSEs/cell/hour (**P = .0078, Wilcoxon; *n* = 8 slices from *N* = 8 animals).

To evaluate the effects of serotonin on individual ARN^KISS^ neuron burst firing and their network activity, 5-HT was applied to acute brain slices prepared from male or diestrous female Kiss1-GCaMP6 mice in which ARN^KISS^ neurons exhibit spontaneous synchronized activity. In this preparation, each calcium transient (event) represents a brief period of burst firing by the recorded ARN^KISS^ neuron (17) and when recording from multiple ARN^KISS^ neurons in the acute brain slice, these transients are often found to align as synchronized episodes termed miniature synchronization episodes (mSEs). These mSEs are thought to be the building blocks of the larger-scale synchronization events occurring in vivo that drive pulsatile LH secretion (17). Coronal brain slices from the middle and caudal ARN were used for GCaMP experiments, with 6 to 20 kisspeptin neurons visible within the focal plane during simultaneous fluorescence imaging. Following a predrug baseline recording (Fig. 3A and 3C), 5-HT (30 µM for 3 minutes) initiated persistent transient activity within the ARN^KISS^ neuronal network. For this reason, a 15-minute analysis window was used to capture this, beginning at the start of 5-HT application.

In diestrous female mice (*n* = 9 slices from 9 animals), individual calcium events (peak ≥ 2 SD above trace mean) occurred at a baseline rate of 5.3 ± 1.2 events/cell/hour and in the presence of 5-HT this rate was 4-fold higher at 22.4 ± 0.9 events/cell/hour (*P* = .0039, W = 45, Wilcoxon test, Fig. 3B). The rate of mSEs (≥2 calcium events occurring within 10 seconds) were also significantly increased with a predrug baseline rate of 0.6 ± 0.2 mSEs/cell/hour and 5-HT increased this 7-fold to 4.2 ± 0.4 mSEs/cell (*P* = .0039, *W* = 45, Wilcoxon test, Fig. 3B). Values “per cell” represent the mean rate at which each neuron takes part in mSEs.

In male mice (*n* = 8 slices from 8 animals), the mean baseline rate of individual calcium events was 8.5 ± 2.3 events/cell/hour, and this more than doubled in the presence of 5-HT to 19.4 ± 3.0 events/cell/hour (*P* = .016, *W* = 34, Wilcoxon test, Fig. 3D). In males, mSEs occurred at a predrug baseline rate of 1.2 ± 0.4 mSEs/cell/hour, and 5-HT increased this 3.5-fold to 4.1 ± 0.7 mSEs/cell/hour (*P* = .0078, W = 36, Wilcoxon test, Fig. 3D).

Individual ARN^KISS^ neurons were defined as “5-HT-responsive” if both (i) the rate of events was ≥3-fold higher in the presence of 5-HT compared with the predrug period for that neuron, and (ii) the post-5-HT (15-25 min) event rate was ≥ 6 events/hour. In males and diestrous females, 77% (51/77 cells from 8 animals) and 76% (57/75 cells from 9 animals) of ARN^KISS^ neurons, respectively, were activated by 5-HT.

**Figure 4:**
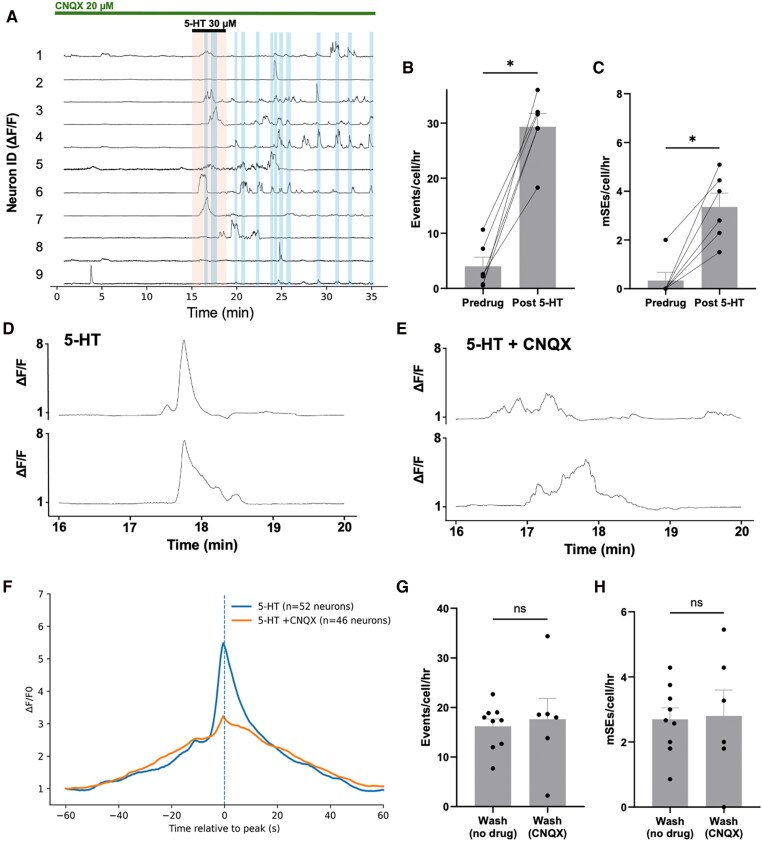
Serotonin-evoked ARN^KISS^ neuron activity persists in the presence of AMPA receptor blockade ex vivo. (A) A representative diestrous female Kiss1-GCaMP6s brain slice, with 9 individual ARN^KISS^ neuron ΔF/F traces recorded simultaneously in the presence of CNQX (20 µM) and a 3-minute exposure to 5-HT (30 µM). Blue shading indicates mini synchronization events (mSEs). (B and C) Histograms showing the effect of 5-HT (30 µM) under continuous CNQX perfusion on mean + SEM calcium events/cell/hour (B) and mSEs/cell/hour (C) (*P = .0313, Wilcoxon; *n* = 6 slices from *N* = 6 animals). (D and E) Representative examples illustrating the profile of 5-HT-evoked transients in the absence (D) and presence (E) of CNQX. (F) Mean event profile of transients in the presence (yellow, *n* = 46 neurons from 6 animals) and absence of CNQX (blue, *n* = 52 neurons from 9 animals). (G and H) Event and mSE rates during the post-5-HT wash period (25-35 minutes)(events *P* = .80; mSEs *P* = .98; Mann–Whitney *U*).

Previous work has shown that glutamate-AMPA receptor signaling is critical for the reciprocally connected ARN^KISS^ neurons to generate synchronized activity (17, 26). As such, we were interested to assess the degree to which interconnected activity amongst ARN^KISS^ neurons may contribute to the high prevalence of excitatory 5-HT responses. To evaluate this, we repeated the 5-HT experiment in slices prepared from 6 diestrous mice but did so in the presence of CNQX (20 µM) to abolish all glutamate-AMPA transmission (Fig. 4A). Under these conditions, 73% of ARN^KISS^ neurons (41/56 cells from 6 animals) were activated by 5-HT. Individual calcium events occured at a baseline rate of 4.0 ± 1.7 events/cell/hour and this was increased 7-fold by 5-HT to 29.3 ± 2.4 events/cell/hour (*P* = .0313, *W* = 21, Wilcoxon test, Fig. 4B). Unexpectedly, the profiles of 5-HT-evoked events were substantially altered in the presence of CNQX with a notable absence of the typical sharp peak in GCaMP fluorescence (Fig. 4D-4F).

As expected, mSE activity was very low or absent in the presence of CNQX occurring at a rate of 0.3 ± 0.3 mSEs/cell/hour but 5-HT increased this to 3.4 ± 0.5 mSEs/cell/hour (*P* = .0313, *W* = 21, Wilcoxon test, Fig. 4C). Further, 5-HT generated an overall identical rate of ongoing activity during the post-5-HT wash period (25-35 m) with and without CNQX. This applied to both the rate of evoked events (16.2 ± 1.5 events/cell/hour no drug; 17.6 ± 4.2 events/cell/hour in CNQX; *P* = .80, *U* = 24.5, Mann–Whitney *U*, Fig. 4G) and mSEs (2.7 ± 0.4 mSE/cell/hour no drug; 2.8 ± 0.8 mSE/cell/hour in CNQX; *P* = .98, *U* = 26.5, Mann–Whitney *U*, Fig. 4H).

### In vivo photometry and serotonin microinfusions into the ARN

**Figure 5:**
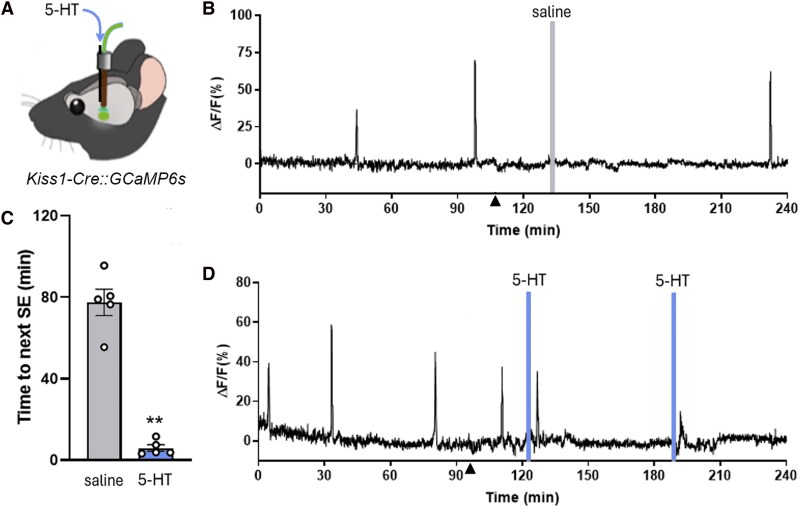
Local infusion of serotonin activates ARN^KISS^ neuron synchronization events. (A) Schematic of experimental approach with 5-HT infusion down a “microfluidic GCaMP optic fiber” assembly directed at the ARN in Kiss1-Cre::GCaMP6s mice. (B) Representative example of ARN^KISS^ neuron population GCaMP activity in a mouse given a saline microinfusion into the ARN. Triangle shows when the injector was placed in the cannula and gray line shows time of 2-minutes saline infusion. (C) Histogram showing mean ± SEM time from saline and 5-HT (10 µM) infusion to the next synchronization episode (SE) (**P < .0001, 2-tailed, unpaired *t*-test: *t*(8) = 10.81, *N* = 5). (D) fiber photometry recording from the same mouse in (B) but now given 2 infusions of 5-HT (blue).

To assess the ability of 5-HT to modulate the kisspeptin pulse generator in vivo, we used a “microfluidic GCaMP fiber photometry” approach that allows the infusion of compounds directly into the ARN while measuring the population activity of ARN^KISS^ neurons in real time (17, 27). Fiber photometry in female diestrous stage Kiss1-cre mice given an AAV9-FLEX-GCaMP6s injection into the ARN revealed the presence of typical ARN^KISS^ neuron synchronization events (SEs) (Fig. 5B). We have previously demonstrated that ∼60% of ARN^KISS^ neurons express GCaMP using this method and this represents over 90% of all GCaMP-expressing cells (22). The ARN^KISS^ neuron SEs recorded with this method are perfectly correlated with pulsatile LH secretion in all mouse models examined to date (20, 22, 28, 29).

After approximately 90 minutes of baseline recording, a fluid injector was inserted into the infusion cannula attached to the optic fiber and, 20-30 minutes later, 1 µM of either saline or 10 µM 5-HT (dissolved in saline) was infused into the ARN over 2 or 10 minutes. Regardless of the infusion duration, this consistently evoked an SE within 3-12 minutes in all 5 mice examined (Fig. 5C and 5D). The mean interval between the infusion of 5-HT and the occurrence of an SE was 6.0 ± 1.6 minutes, whereas the time to the next SE following saline infusion controls was 77.4 ± 6.4 minutes (P** < .0001, 3-tailed, unpaired *t*-test: *t*(8) = 10.81.) (Figure 5C). In 3 mice, a second infusion of 5-HT was possible, and this was also found to generate an ARN^KISS^ neuron SE. Presumably due to receptor desensitization, the amplitude of the second evoked SE was 0.54 ± 0.12% of the first SE (Fig. 5D).

## Discussion

The dorsal and median raphe serotonergic neurons innervating the forebrain exist as functionally heterogeneous subpopulations that regulate multiple adaptive responses (1, 30). The precise identity of the raphe subpopulation innervating the ARN^KISS^ neurons is not known but approximately 5% of dorsal raphe serotonin neurons innervate the ARN (31) and these cells are likely to be part of the subtype co-expressing GABA and thyrotropin-releasing hormone (32). We show here that the activation of 5-HT receptors on ARN^KISS^ neurons, reflecting the activation of this brainstem input, results in enhanced excitability, bursting behavior, and synchronicity within the population. As ARN^KISS^ neuron population synchronizations drive each pulse of LH, this indicates that serotonergic inputs can provide a potent facilitatory influence on the frequency of pulsatile reproductive hormone secretion.

We find that around 60%-70% of ARN^KISS^ neurons are activated by 5-HT while less than 10% of the population are inhibited. These heterogeneous responses to 5-HT are typical in the forebrain and, indeed, Buo et al (33) similarly found that ∼50% of the surge generator kisspeptin neurons located in the rostral periventricular area of the third ventricle (RP3V) are activated by 5-HT while <10% are inhibited. We observed that ∼60% of ARN^KISS^ neurons were activated by short puffs of 5-HT while ∼70% exhibited increases in GCaMP fluorescence following bath exposure of 5-HT. These stimulatory effects are direct as, alongside the expression of multiple Htr transcripts in ARN^KISS^ neurons, these cells are activated by 5-HT in the presence of TTX and a cocktail of amino acid receptor antagonists.

An interesting feature of the 5-HT response is the way in which ARN^KISS^ neurons exhibit continued transient activity for several minutes beyond their exposure to serotonin. This may result from intrinsic mechanisms in which 5-HT-activated signaling cascades within ARN^KISS^ neurons continue to operate beyond their initial activation to keep generating bursts of firing. While not typical of all neurotransmitters activating ARN^KISS^ neurons, this pattern of extended oscillation has also been observed with vasoactive intestinal peptide (34). Another possibility is that these extended activations in ARN^KISS^ neurons result from the generation of reverberating network activity. The ARN^KISS^ neurons are thought to utilize glutamate-AMPA receptor transmission amongst their collateral connections to enable synchronization (17). However, this seems not to be the case, as the on-going transient activity evoked by 5-HT continued in the presence of CNQX. Interestingly, however, we did detect that the events evoked by 5-HT in the presence of CNQX were notably flattened without the normal sharp peak in signal. This demonstrates that the final intense period of firing that drives this calcium peak is dependent on glutamate transmission. Whether this occurs from glutamate-AMPA signaling amongst network collaterals or recurrent collaterals of the cells themselves remains to be examined.

Serotonin typically activated ARN^KISS^ neurons within tens of seconds when puffed directly in the vicinity of their cell bodies, 2-3 minutes when included into the perifusion medium in ex vivo calcium imaging experiments, and 3-12 minutes when infused into the ARN in vivo. These temporal differences likely reflect the time required for diffusion of 5-HT through increasingly complex and dense neuropils to reach an ARN^KISS^ neuron or a sufficient number of kisspeptin neurons to evoke an SE. A normal synchronization event involves over 85% of the ARN^KISS^ neuron population (17, 35). It is thought that mSEs represent synchronizations amongst small numbers of ARN^KISS^ neurons that have failed to recruit sufficient neurons to generate the all-or-nothing explosive SE (17). Presumably due to the low numbers of <25 ARN^KISS^ neurons recorded at any one time in the brain slice, full SEs are never seen in this preparation. The observation here that 5-HT evokes ongoing mSEs ex vivo suggests that this would provide a powerful stimulus promoting the occurrence of large-scale synchronizations leading to an SE in vivo.

Our receptor pharmacology analysis suggests a complex pattern of 5-HT receptor subtypes mediating the effects of serotonin on ARN^KISS^ neurons. The transcriptomic analysis demonstrates the presence of low variable levels of Htr1a, 1b, 1d, 2a, 2b, 2c transcripts with more robust expression of 4, 5a, 6, and 7 mRNAs in ARN^KISS^ neurons from intact diestrous mice. As 5-HT_5_ receptors are exclusively inhibitory (36), they may underlie the ∼10% of ARN^KISS^ neurons that are inhibited by serotonin. However, the majority of the population excited by serotonin appear to be using different combinations of 5-HT receptors. Of these, one-third have the stimulatory effects of serotonin blocked by methiothepin, a broad-spectrum antagonist at 5-HT_1,2,5,6,7_ receptors. This indicates that this third of serotonin-stimulated ARN^KISS^ neuron population use 5-HT_1_, 5-HT_2_, 5-HT_6_, and/or 5-HT_7_ receptors. As Htr3 transcripts are not found in ARN^KISS^ neurons, the remaining two-thirds of neurons not affected by methiothepin may possibly use 5-HT_4_ receptors. Gocz et al (13) reported that 5-HT_4_ receptors were primarily responsible for the stimulatory effects of serotonin on ARN^KISS^ neuron excitability in slices from estrogen-replaced OVX mice. We note in our own studies, that a 5-HT_4_ receptor agonist did not activate ARN^KISS^ neuron firing but did, nevertheless, greatly facilitate the effects of serotonin. Thus, we find that the 5-HT_4_ receptor is not sufficient on its own to activate ARN^KISS^ neurons in diestrous mice. Brought together, this suggests that the co-activation of multiple 5-HT receptors is likely required for the full stimulatory effect of serotonin on the ARN^KISS^ neuron population.

The proposed mode of combinatorial 5-HT receptor activation in ARN^KISS^ neurons may explain why a 5-HT_2_ receptor antagonist was not effective on its own in blocking the stimulatory effects of serotonin. However, this is in contrast to studies in the goat where the stimulatory effects of ICV serotonin on the pulse generator activity were completely abolished by a 5-HT_2_ receptor antagonist (9). It is possible that serotonin given into the ventricular system acts on 5-HT_2_ receptors outside the ARN to indirectly modulate ARN^KISS^ neurons in the goat (9). However, Htr2 was the only serotonin receptor transcript identified in rat ARN^KISS^ neurons (9) and this is in marked contrast to the mouse.

Early studies found that 5-HT enhanced GnRH secretion from hypothalamic explants taken from estradiol-treated OVX rats and that 5-HT receptor antagonists abolished pulsatile LH secretion in OVX guinea-pigs (7, 8). Recently, the central administration of 5-HT was observed to evoke an ARN MUA volley in the OVX goat (9). These observations are all compatible with a direct stimulatory effect of 5-HT on the ARN^KISS^ neuron pulse generator. It remains, however, that ICV administration of 5-HT has been found to consistently suppress pulsatile LH secretion in OVX rats (3, 4). There are at least 3 explanations for the apparent inhibitory effects of 5-HT on LH pulsatility. First, ICV serotonin will modulate the activity of most neuronal circuitries and some of these may provide inputs to the ARN^KISS^ neurons. Second, it is possible that the removal of gonadal steroids has a major impact on the way in which 5-HT modulates ARN^KISS^ neuron activity. However, Gocz et al (13) demonstrated that estradiol suppressed the 5-HT activation of ARN^KISS^ neuron; an action contrary to the observation that OVX switches excitation to inhibition. Third, it is important to note that many neurotransmitters activate LH release in the presence of estradiol whilst suppressing LH in OVX animals (5). Thus, this behavior is dependent upon the state of the animal rather than any specific transmitter. It was found that the same level of activation of ARN^KISS^ neurons can stimulate or collapse pulsatile LH secretion depending on whether the pulse generator is operating at a low or high frequency, respectively (6). Thus, it is possible that the 5-HT activation of the pulse generator when operating at its maximum frequency in the OVX state results in the suppression of pulsatile LH secretion.

These observations indicate that ascending serotonergic neurons provide a direct excitatory input to ARN^KISS^ neurons that facilitate pulse generator frequency in both male and female mice. However, the functional importance of this input remains unknown. Studies have shown that the SSRI fluoxetine can partially restore LH pulsatility in hyperglycemic rats (9) and also rescue estrous cyclicity in fasted mice (10). While this demonstrates that increasing serotonin levels can activate the ARN^KISS^ neuron pulse generator in the face of different negative modulators of fertility, it does not identify serotonin as mediating stress responses in this circuit.

It is also interesting to consider the role of 5-HT in the broader context of the hypothalamic network controlling GnRH secretion. Serotonin is known to exert short duration inhibitory and longer duration excitatory actions on the GnRH neuron cell body (37) and predominant excitatory actions on the RP3 V kisspeptin surge generator neurons (33). These inputs to the GnRH neuron cell body and RP3V^KISS^ neurons would be expected to modulate GnRH surge generation (38, 39). Thus, although it is unknown if the same brainstem serotonin neuron populations innervate GnRH neurons, RP3V kisspeptin, and ARN^KISS^ neurons, the net outcome of such activation would facilitate both LH surge and pulse generation in females.

In summary, we show here that 5-HT exerts a direct stimulatory influence on the excitability of ARN^KISS^ neurons through the likely complex and heterogeneous involvement of different 5-HT receptors. This results in increased burst firing and increased ARN^KISS^ neuron population synchronicity resulting in the generation of synchronization episodes responsible for LH pulses. While this evidence, alongside that of other investigators, clearly identifies ascending serotonergic neurons as positive regulators of ARN^KISS^ neuron pulse generator frequency in both sexes, their physiological roles remain unclear.

## Data Availability

All datasets generated during and/or analyzed during the current study will become publicly available on the University of Cambridge Apollo Repository and will also be available from the corresponding author.
